# Comparative proteomic analysis of malformed umbilical cords from somatic cell nuclear transfer-derived piglets: implications for early postnatal death

**DOI:** 10.1186/1471-2164-10-511

**Published:** 2009-11-05

**Authors:** Jong-Yi Park, Jae-Hwan Kim, Yun-Jung Choi, Kyu-Chan Hwang, Seong-Keun Cho, Ho-Hyun Park, Seung-Sam Paik, Teoan Kim, ChanKyu Park, Hoon Taek Lee, Han Geuk Seo, Soo-Bong Park, Seongsoo Hwang, Jin-Hoi Kim

**Affiliations:** 1 Animal Resource Research Center, College of Animal Bioscience and Technology, KonKuk University, Seoul 143-701, South Korea; 2 Department of Biomedical Science, College of Life Science, CHA University, Pochon-si, Gyeonggi-do 487-010, South Korea; 3 CHO-A Biotechnology Research Institute, CHO-A Pharmaceutical Co. Ltd., Seoul 150-992, South Korea; 4 Department of Pathology, College of Medicine, Hanyang University, Seoul 133-791, South Korea; 5 Department of Physiology, Catholic University of Daegu School of Medicine, Daegu 705-718, South Korea; 6 Department of Pharmacology, Gyeongsang National University School of Medicine, Jinju, GyeongNam 660-701, South Korea; 7 Animal Biotechnology Division, National Institute of Animal Science, Rural Development Administration, Suwon 441-706, South Korea

## Abstract

**Background:**

Somatic cell nuclear transfer (scNT)-derived piglets have high rates of mortality, including stillbirth and postnatal death. Here, we examined severe malformed umbilical cords (MUC), as well as other organs, from nine scNT-derived term piglets.

**Results:**

Microscopic analysis revealed complete occlusive thrombi and the absence of columnar epithelial layers in MUC (scNT-MUC) derived from scNT piglets. scNT-MUC had significantly lower expression levels of platelet endothelial cell adhesion molecule-1 (PECAM-1) and angiogenesis-related genes than umbilical cords of normal scNT piglets (scNT-N) that survived into adulthood. Endothelial cells derived from scNT-MUC migrated and formed tubules more slowly than endothelial cells from control umbilical cords or scNT-N. Proteomic analysis of scNT-MUC revealed significant down-regulation of proteins involved in the prevention of oxidative stress and the regulation of glycolysis and cell motility, while molecules involved in apoptosis were significantly up-regulated. Histomorphometric analysis revealed severe calcification in the kidneys and placenta, peliosis in the liver sinusoidal space, abnormal stromal cell proliferation in the lungs, and tubular degeneration in the kidneys in scNT piglets with MUC. Increased levels of apoptosis were also detected in organs derived from all scNT piglets with MUC.

**Conclusion:**

These results suggest that MUC contribute to fetal malformations, preterm birth and low birth weight due to underlying molecular defects that result in hypoplastic umbilical arteries and/or placental insufficiency. The results of the current study demonstrate the effects of MUC on fetal growth and organ development in scNT-derived pigs, and provide important insight into the molecular mechanisms underlying angiogenesis during umbilical cord development.

## Background

In the past decade, several species of animal, including goat, pig, sheep and cattle, have been cloned using scNT techniques [1]. However, while cloning is widely used in basic research, as well as in some biomedical and agricultural applications, a number of substantial problems exist with the current technologies, including relatively low success rates and severe defects of the fetus and/or placenta resulting in abortion, neonatal death and postnatal disease [2-4]. It has been suggested that a major cause of early fetal loss of cloned animals is the high incidence of placental abnormalities that can occur throughout the entire gestation period.

Studies have shown that aberrant methylation in the trophetoderm of cloned blastocysts can induce global gene dysregulation in extra-embryonic regions, and that this type of gene dysregulation can potentially lead to the development of a dysfunctional placenta and have a detrimental effect on overall fetal development [5-7]. It has long been recognized that the major limitation of animal cloning with regard to efficient animal production relates to inadequate conceptus--maternal interactions through the placenta [8]. This inadequacy leads to deficiencies in fetal growth *in utero*, which can affect the survival, health and well-being of the newborn and the productivity of the animal at adulthood. Inadequate conceptus-maternal interaction is particularly acute in animal production using scNT. The incidence of abnormal placental development is very high in scNT-derived animals, and is largely responsible for the frequent fetal loss, late-term pregnancy complications and perinatal mortality associated with this technique [9]. Recently, we were able to successfully clone piglets using scNT, but the procedure resulted in high level of phenotypic abnormalities that compromised fetal and postnatal health [3,7,10,11]. As a result, the birth rate and postnatal survival of the cloned piglets was very low. Subsequently, we determined that the low birth rate of the cloned animals was due largely to abnormal levels of apoptosis in extraembryonic tissue during early pregnancy [3,12] and/or placental insufficiency at term [11].

A number of human umbilical cord malformations have been documented, most of which involve blood vessels [13]. One such arterial malformation, human single umbilical artery (SUA) syndrome, in which one of the two umbilical arteries is absent in the cord, is believed to be caused by insufficient irrigation of the terminal portion of the embryo. SUA syndrome affects 0.2-1% of pregnancies, and the clinical symptoms vary depending on the stage of development at which it occurs [14]. Hypoplasia of one of the two umbilical arteries also occurs, albeit much less frequently (0.03% of pregnancies). Hypoplastic umbilical arteries (HUAs) are defined as malformed arteries that exhibit an artery-artery diameter difference of approximately 50%. HUAs are associated with pre/perinatal morbidity and congenital abnormalities, and have also been linked to intrauterine growth retardation, maternal diabetes, polyhydramnios and congenital cardiopathy [15]. The development of HUAs most likely represents an incomplete form of SUA syndrome [16].

Of the 65 term piglets generated by scNT, we observed the high incidence of umbilical cord malformation (9/65, 13.9%), which led us to speculate that piglets with MUC might be a good model for studying human HUA syndrome. In the current study, we demonstrated that scNT piglets with MUC exhibit impaired endothelial cell migration and angiogenesis, defects that might underlie the development of HUA and/or placental insufficiency in these animals. We also analyzed gene and protein expression patterns in the umbilical cord to identify putative umbilical cord markers (either proteins or protein modifications) that were associated with poor outcomes for the fetus. Using this approach, we can begin to elucidate the effects of MUC on fetal growth and organ development in scNT-derived pigs, and to investigate the molecular mechanisms of angiogenesis during umbilical cord development.

## Methods

Animals were maintained and experiments were conducted in accordance with the Kon-Kuk University Guide for the Care and Use of Laboratory Animals.

### Somatic cell nuclear transfer and embryo transfer

Nuclear transfer was carried out as described in previous reports [10,11,17]. Briefly, the matured eggs with the first polar body were cultured in medium supplemented with 0.4 mg/ml demecolcine (Sigma) and 0.05 mol/l sucrose for 1 hour (hr). Sucrose was used to enlarge the perivitelline space of the eggs. Treated eggs with a protruding membrane were moved to medium supplemented with 5 mg/ml cytochalasin B and 0.4 mg/ml demecolcine and the protrusion was removed with a beveled pipette. A single donor fetal fibroblast cell derived from gestational day 30-derived Duroc, Berkshire and 3 way hybrid (Landrace × Duroc × Yorkshire) fetus was injected into the perivitelline space of each egg and electrically fused using two direct current pulses of 150 V/mm for 50 msec in 0.28 mol/L mannitol supplemented with 0.1 mM MgSO4 and 0.01% polyvinyl alcohol (Sigma). Fused eggs were cultured in medium with 0.4 mg/ml colcemid for 1 h before parthenogenetic activation, and then cultured in 5 mg/ml of CB-supplemented medium for 4 h. The reconstructed oocytes were activated by 2 direct current pulses of 100 V/mm for 20 msec in 0.28 mol/L mannitol supplemented with 0.1 mmol/L MgSO_4_, and 0.05 mmol/L CaCl_2_. Activated eggs were cultured in the medium for 6 days in an atmosphere of 5% CO_2 _and 95% air at 39°C.

Gilts (Duroc × Yorkshire × Landrace) of at least eight months of age were used as recipients. Estrus synchronization of recipients was carried out as reported previously [10,11]. ScNT embryos were surgically transferred into oviducts of synchronized recipients. The pregnancy status of recipients was determined by ultrasound between days 30-35. Recipients produced scNT-derived piglets via vaginal delivery.

Umbilical cord samples obtained from three control piglets and 65 scNT-derived piglets were washed with PBS and used for the purification of endothelial cells or analysis of gene and protein expression. scNT-N samples with normal phenotype after 6 month of birth were selected by comparing with control groups and used for further experiments.

### Isolation and characterization of porcine umbilical vein-derived endothelial cells (PUVEC)

PUVEC were obtained from MUC (designated as scNT-MUC) and normal (designated as scNT-N) umbilical cords from scNT piglets, or normal umbilical cords from piglets derived by artificial insemination (designed as control), as described previously [18]. PUVEC were grown until they reached confluence (between 3 to 5 days), and were re-fed every 2 to 3 days by exchanging half of the growth medium with fresh medium. Alternatively, PUVEC were stored in liquid nitrogen until use. The purity of each PUVEC population was confirmed by immunostaining with an anti-PECAM-1 (CD31) antibody (Santa Cruz Biotechnology, Santa Cruz, CA, USA) and counterstaining with 4',6-diamidino-2-phenylindole (DAPI; 1:15,000, Sigma, St. Louis, MO).

### RNA isolation and real time reverse transcriptase (RT)-PCR

Total RNA was extracted from umbilical cord tissue using a Micro-to-Midi Total RNA Purification System (Life Technologies Inc., Carlsbad, CA, USA). Real-time RT-PCR was conducted using a DNA Engine OPTICON2 system (MJ Research, San Francisco, CA, USA) and SYBR Green as the double-stranded DNA-specific fluorescent dye (SYBR Green qPCR premix, FINNZYMES, Woburn, MA, USA). Target gene expression levels were normalized to tubulin gene expression, which was unaffected in scNT-derived pigs. The RT-PCR primer sets are shown in Additional file 1. Real-time RT-PCR was independently performed in triplicate for different samples and the data were expressed as the mean value of gene expression measured in individual control, scNT-N and scN-MUC, using each individual animal as experimental unit.

### Endothelial cell (EC) migration/motility assay

Migration assays were performed as described by Rudolph *et al *[19], with slight modifications. PUVEC were grown in 35 mm culture dishes to 80% confluence. A wound was formed by clearing the monolayer from an area of the dish with a 200 μl pipette tip. The boundary of the wound was marked and the cells were allowed to incubate for 24 hr. The cells were fixed, stained with a 1:10 dilution of Giemsa (Sigma), and then photographed. Cell migration was measured by counting the number of cells that migrated into the cleared area of the dish. Data represents the means of four different random fields.

### Tubule formation assay

Matrigel-sandwich tubule formation assays were performed as described previously [16,20]. Briefly, cold Matrigel solution was added to a 96-well plate and allowed to air-dry. Following rehydration of the Matrigel, ECs derived from control, scNT-N or scNT-MUC were seeded onto the Matrigel at a density of 10,000 cells per well, and then allowed to incubate at 37°C for 24 hr. Microtubules were visualized by microscopy and photographed using a digital camera.

### 2-dimensional gel electrophoresis (2-DE)

2-DE analysis was performed by using 3 controls, 3 scNT-N, and 6 scNT-MUC samples with three replicates independently. Umbilical cords were solubilized in lysis buffer containing 7 M urea, 2 M thiourea, 4% w/v CHAPS, 40 mM DTT and 0.5% Pharmalyte pH 4-7. Insoluble material was removed by centrifugation. IPG strips (17 cm, pH 4-7, Bio-Rad, Hercules, CA, USA) were rehydrated overnight in 300 μL of lysate containing 500 μg of protein. Isoelectric focusing was performed using a Protein IEF Cell (Bio-Rad). The focused strips were then equilibrated by incubating them first in equilibration solution (6 M urea, 30% v/v glycerol, 2% w/v SDS, 50 mM Tris-Hcl, pH 8.8) containing 1% w/v DTT for 15 min, followed by a second incubation in 2.5% w/v iodoacetamide in the same equilibration solution for 15 min. 2-DE was performed using 0.7 cm thick, 18 × 18 cm linear gradient gels (7.5-17.5%) in a Protein ∏ xi 2-D Cell apparatus (Bio-Rad). The gels were stained with silver or Coomassie Brilliant Blue G250 to visualize proteins. Images of stained gels were digitized with a densitometer (Versa Doc Imagin System 1000™, Bio-rad). The density of spots was detected and counted by both automation and manual spot-detection, and statistically analyzed with PDQuest software (Version 7.1.1, Bio-Rad). Protein expression data from gels were normalized for the total density presented in gel images. Protein identification procedure was described in detail in Additional file 2.

### Western blot analysis

Western blot analysis was performed as described previously [3,11]. Molecular weight standards were obtained from New England Biolabs (Ipswich, MA, USA). Membranes were probed with primary antibodies recognizing the following proteins: SOD-Cu/Zn, SOD-Mn, and aldose reductase were gifts from Dr. Seo H-G (Gyeongsang National University, Jinju, South Korea). peroxiredoxin-2, -4, Bax and Annexin-A5, HSP-27 antibodies were purchased from Santa Cruz (CA, USA). Active- and pro-caspase-3, active- and pro-caspase-8, active PARP was from MERK (Darmstadt, Germany). Bcl-2 and actin antibody were from Abcam (Cambridge, UK) and CHEMICON (Temecula, CA, USA), respectively. Thereafter, the membranes were incubated with an appropriate horseradish peroxidase-conjugated secondary antibody (Jackson Immunoresearch, West Grove, PA, USA) and subjected to enhanced chemiluminescence analysis (Amersham, Piscataway, NJ, USA). An anti-actin antibody (1:500, CHEMICON International) was used to verify equal protein loading. Signals were visualized by using the ECL kit (Amersham). Band intensities of each protein expression were quantified by Image processing and analysis using Image J 1.23 (NIH image).

### TdT-mediated dUTP-X Nicked End labeling (TUNEL) assay

TUNEL assays were performed as described previously [21]. Briefly, tissue sections were incubated with TUNEL mix (0.3 U/μL calf thymus terminal deoxynucleotidyl transferase, 7 pmol/μL biotin dUTP, 1 mM cobalt chloride in 1 × reaction buffer in distilled water) and then washed. Sections were saturated and then treated with a 1:20 dilution of ExtraAvidin peroxidase antibody. After washing, sections were incubated in DAB staining solution [1.24 mg DAB, 25 μL 3% NiCl_2_, 152 μL 1 M Tris-HCl (pH 7.5) in 2 ml distilled water]. Slides were mounted in crystal mount (Biomeda, Foster City, CA, USA) and visualized by microscopy.

### Statistics

All experimental data represent the means ± standard deviation (SD). Analysis of angiogenesis-related gene expression pattern in figure two were performed three times by using 10 scNT-N and 6 scNT-MUC piglets and the statistical significance was confirmed by using t-test. Wound healing and tubule formation in figures five and six were analyzed by using 2 controls, 3 scNT-N and 6 scNT-MUC samples with three replicates and the statistical significance was calculated by ANOVA and confirmed by Duncan's multiple range procedure. Protein and mRNA expression levels (figure seven) were examined in 3 controls, 6 scNT-MUC with three replicates and the statistical significance was confirmed by t-test.

## Results and discussion

### High incidence MUC in scNT-derived piglet clones

In this study, we carried out scNT using each donor fibroblast cell derived from Duroc, Berkshire and 3 way hybrids (Landrace × Duroc × Yorkshire). scNT embryos were transferred and successfully produced 65 cloned piglets from 24 surrogates (Table 1). The number of offsprings was variable depending on the surrogates ranging from 1 to 6 piglets. Genetic identification for scNT clones was confirmed by using porcine DNA microsatellite markers (SWR1120, SW1311, and SW1327) (data not shown). Of these 9 piglets were derived from Duroc, 12 piglets from 3 way hybrid, and 44 piglets from Berkshire: among them, 14 piglets (21.5%) were stillborn, 38 scNT piglets (58.5%) died suddenly within the first week of life, and 3 piglets (4.6%) died during puberty. To date, only 10 scNT pigs (4 piglets were derived from Duroc, 3 piglets from 3 way hybrid, and 3 clones from Birkshire) are still alive and healthy. Analysis of 65 term scNT-derived piglet clones revealed that nine had severe MUC, a prevalence of 13.9%. Of the 56 scNT piglets with normal umbilical cords, twelve (21.4%) were stillborn, 33 (58.9%) died suddenly within the first week of life, and one (1.8%) died during puberty. Ten (17.9%) of the 56 scNT-N pigs survived into adulthood, and remain alive and healthy. Of the nine scNT piglets with MUC, two (22.2%) were stillborn and four died within the first week of life. Of the remaining scNT-MUC piglets, two reached puberty but then died between 8-12 months of age. Only one of the nine scNT-MUC piglet clones survived to adulthood. The birth weight (0.882 ± 0.243 kg; n = 9) and placenta weight (0.24 ± 0.04 kg; n = 9) of scNT-MUC clones were significantly lower (p < 0.05) than those of scNT-N clones (1.390 ± 0.675 kg and 0.28 ± 0.05 kg, respectively; n = 10) and control piglets [1.352 ± 0.112 kg (n = 20) and 0.33 ± 0.08 kg (n = 35), respectively]. Further analyses were carried out by using Berkshire-derived scNT clones and controls to minimize breeder variability.

**Table 1:** Efficacy of scNT-derived piglet production

Group	Embryo transferred	Recipients	Pregnancy (%)	Abortion (%)*	Delivery (%)	Piglets (%)**	Gestational length (days)
scNT	62,090	265	57 (21.5)	33 (57.9)	24 (42.1)	65 (0.1245%)	118.4 ± 2.408
Artificial insemination	-	12	12	0	12 (100)	144	114.5 ± 0.7

* Abortion (%) presents the percentage of aborted recipients (33) out of pregnant ones (57) at gestational day 30.

** Production efficiency of scNT-derived piglet per transferred embryo.

**Figure 1:**
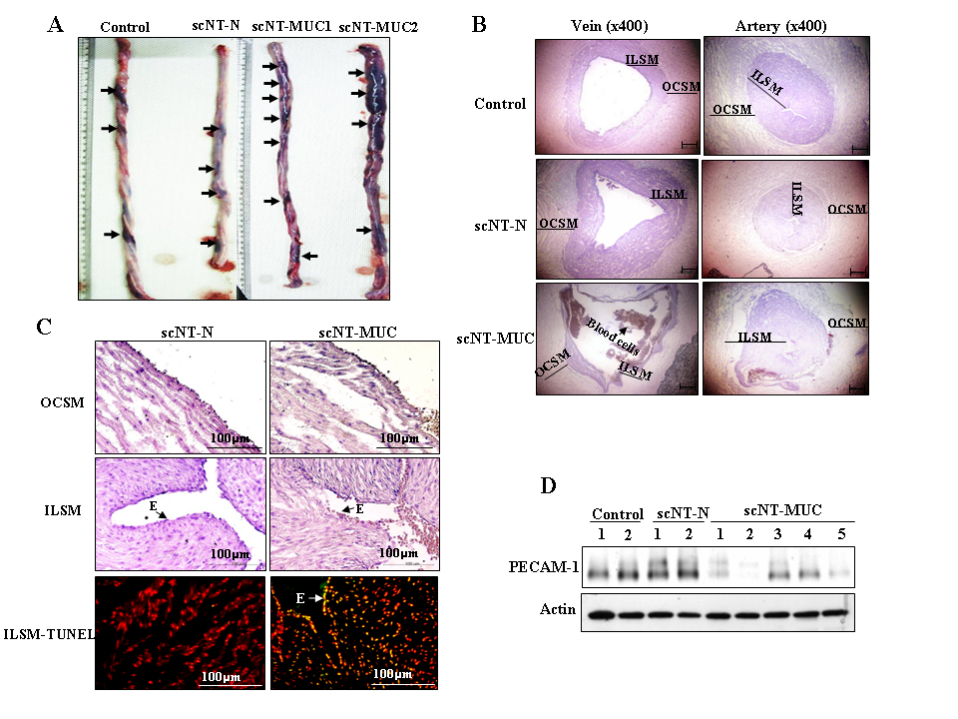
**Morphological and histological analysis of control, scNT-N and scNT-MUC term piglets**. A) scNT-MUC exhibited more swelling and severe damage as compared to control and scNT-N. Of particular interest, the tissues of scNT-MUC were extremely fragile as compared to control and scNT-N. B) Hematoxylin and eosin staining of umbilical vein and artery sections from control, scNT-N and scNT-MUC. The veins and arteries of scNT-N exhibited normal development, whereas in scNT-MUC, they were relatively less mature. C) Regions of ILSM and OCSM in scNT-MUC exhibited severe damage. The OCSM was irregular and/or intermittently dense, and the ILSM had unclear boundaries as compared to scNT-N. The ILSM and epithelium (E) of scNT-MUC (right bottom panel) also had more apoptotic cells than scNT-N (left bottom panel). Representative TUNEL assays are shown. D) Western blot analysis of control, scNT-N, and scNT-MUC using anti-PECAM-1 antibodies. β-actin was analyzed as a control.

Upon histological examination, scNT-MUC exhibited severe tissue damage as compared to control and scNT-N, including tissue swelling (Figure 1A, arrows). Microscopic analysis revealed complete occlusive thrombi in the veins and arteries of scNT-MUC, but not in control and scNT-N (Figure 1B). scNT-MUC also exhibited areas of irregular outer circular smooth muscle (OCSM) and unclear boundaries in the inner longitudinal smooth muscle (ILSM) (Figure 1C). Analysis of umbilical cords using the TUNEL assay to detect apoptotic nuclei clearly showed that there was a high level of apoptosis in the ILSM of scNT-MUC, whereas apoptotic cells were rarely detected in the ILSM of scNT-N umbilical cords (Figure 1C; bottom panel). These results suggested that there is severe vessel damage to both veins and arteries in scNT-MUC.

When we examined the expression of PECAM-1 (CD31), which is abundantly expressed in ECs [22], scNT-MUC expressed significantly lower levels of PECAM-1 than control and scNT-N (Figure 1D). PECAM-1 is an efficient signaling molecule that functions in diverse aspects of vascular biology, including angiogenesis, platelet function and the regulation of leukocyte migration [23]. Thus, impaired expression of PECAM-1 in the umbilical cord could be a contributing factor in the development of umbilical cord malformation.

**Figure 2:**
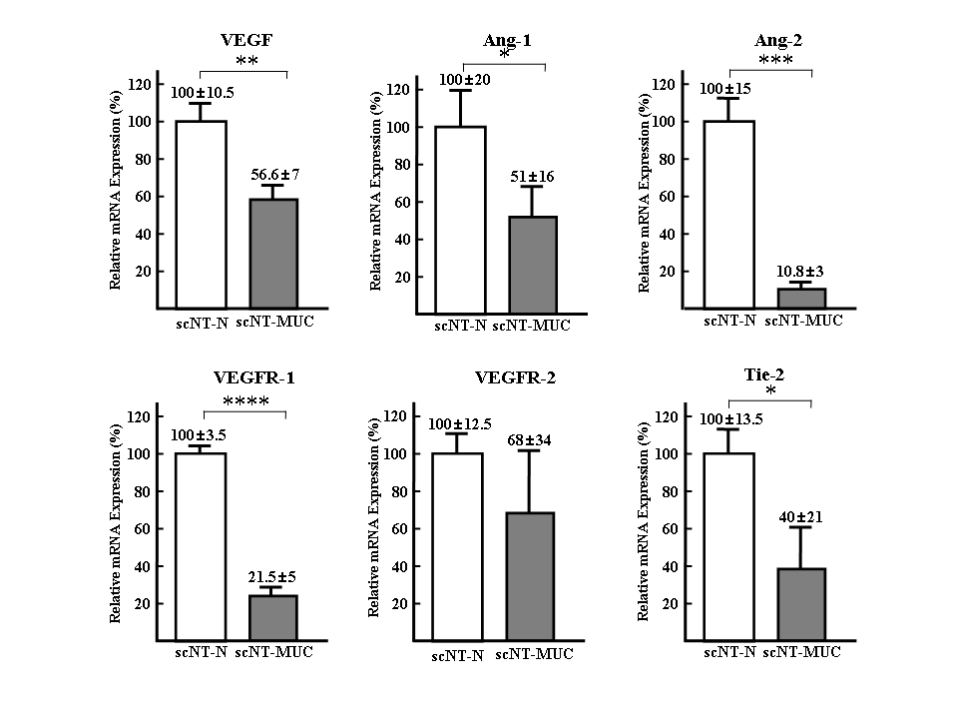
**Angiogenesis-related gene expression in scNT-N and scNT-MUC**. Real-time RT-PCR was carried out using primers that were specific for VEGF, Ang-1 and Ang-2, and their cognate receptors VEGFR-1, VEGFR-2 and Tie-2, respectively. Real-time RT-PCR was independently performed in triplicate for different samples and the data were expressed as the mean value of gene expression measured in individual scNT-N and scN-MUC. Gene expression levels were normalized to tubulin gene expression, and are expressed relative to scNT, which was set as 100 (%). * *p *< 0.05, ** *P *< 0.01, *** *P *< 0.005, **** *P *< 0.001

EC activation is initiated by the binding of proangiogenic factors, such as vascular endothelial growth factor (VEGF), to their cognate receptors on endothelial cells, an event that induces the activation of angiogenic signaling pathways [24]. VEGF is one of the most potent inducers of vascular permeability, and specifically targets ECs through binding to specific endothelial cell surface receptors, including VEGFR-1 and VEGFR-2. The activation of VEGFR-1 induces endothelial permeability [25], while VEGFR-2, which is expressed exclusively in ECs, appears to play a pivotal role in EC differentiation and vasculogenesis [26]. Angiopoietin-1 (Ang-1), a key molecule in the regulation of embryonic vascular development, binds to and activates the endothelial specific receptor Tie2, while Ang-2 is required for subsequent postnatal vascular remodeling [27]. scNT-MUC had significantly lower mRNA expression levels of VEGF, Ang-1, Ang-2, VEGFR-1 and Tie-2 (Figure 2), as compared to scNT-N umbilical cords. The expression of VEGFR-2 was also lower in scNT-MUC than scNT-N umbilical cords. These results suggested that ECs in scNT-MUC fail to elicit mitogenic responses and/or form tubules due to the down-regulation of vascular signaling molecules (i.e. VEGF, angiopoietin and/or their cognate receptors) that play essential roles in vascular development. These defects in EC signaling might be responsible, at least in part, for the developmental abnormalities in scNT-MUC.

**Figure 3:**
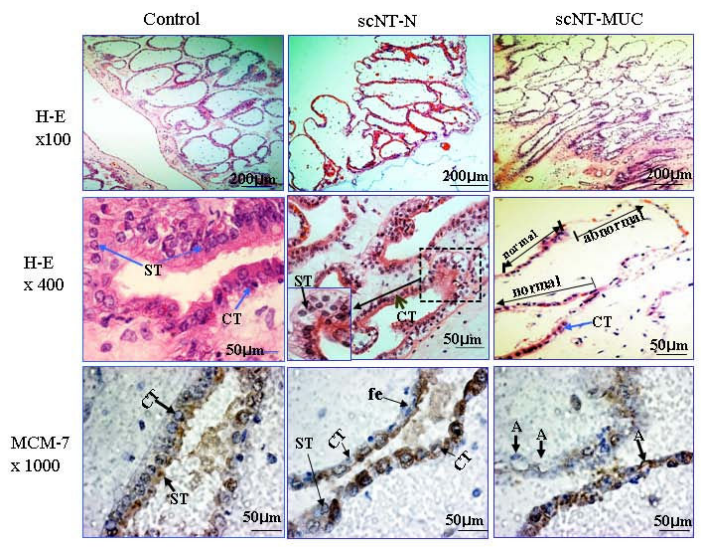
**Photomicrographs of cross-sections of control, scNT-N- and scNT-MUC-derived term placentas**. Dewaxed paraffin sections were stained with hematoxylin and eosin. The villi and column trophoblast cells developed normally in control term placentas, but were poorly developed in scNT-N and scNT-MUC placentas. Immunohistochemical analysis of MCM-7 expression (*lower panels*). In control and scNT-N-derived term placentas, there was extensive MCM-7-positive immunoreactivity in cytotrophoblast (CT) and syncytiotrophoblast (ST) cells, which are located along the basement membrane layer of the branched villi, as well as fetal cells (fe). In contrast, MCM-7 was rarely detected in the anchoring villi of scNT-MUC-derived placentas. MCM-7-positive cells in scNT-MUC-derived placentas exhibited severe damage (A: aponecrosis, arrow).

We also observed several abnormalities in scNT-MUC-derived placentas, including villous hypovascularity and cytotrophoblast and syncytotrophoblast hypoplasia (Figure 3). Placentas derived from scNT-N exhibited similar abnormalities (Figure 3, middle column), however, there were some subtle differences between scNT-MUC- and scNT-N-derived placentas (Figure 3, right column). Most scNT-MUC-derived placentas had fewer blood vessels than normal placentas (data not shown), and exhibited severe aponecrosis of the cytotrophoblast and syncytotrophoblast cells in the developing villi (Figure 3, lower right column). In placentas in which these cells were intact, there was a significant decrease in the expression of the cytotrophoblast biomarker MCM-7 (Figure 3, bottom section). These observations suggested that the placenta abnormalities in the scNT-MUC clones may be associated with a functional abnormality of the ECs and that this may affect the size and survival of the developing fetus.

### Umbilical-derived vein endothelial cells of scNT-MUC clones exhibit abnormal tubular junctions and tubule formation

**Figure 4:**
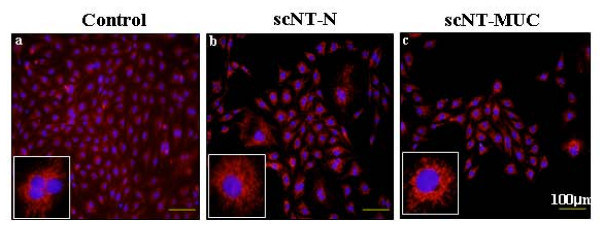
**Immunohistochemical analysis of ECs derived from control (a), scNT-N (b), and scNT-MUC (c)**. Cells were cultured *in vitro *and then stained using an anti-CD31 (PECAM-1, red) antibody, followed by counterstaining of nuclear DNA with DAPI (blue). Insets show individual cells.

PUVECs were obtained from the veins of control, scNT-N and scNT-MUC, as described previously [18]. We established five cell lines from nine scNT-MUC piglets, and determined the purity of each PUVEC line by immunostaining using anti-PECAM-1 (CD31) or anti-Factor Vlll (VWF) antibodies (data not shown). As shown in Figure 4, most of the cells derived from control, scNT-N and scNT-MUC piglets were positive for CD31, which indicated that they are umbilical-derived EC lines of relatively high purity.

**Figure 5:**
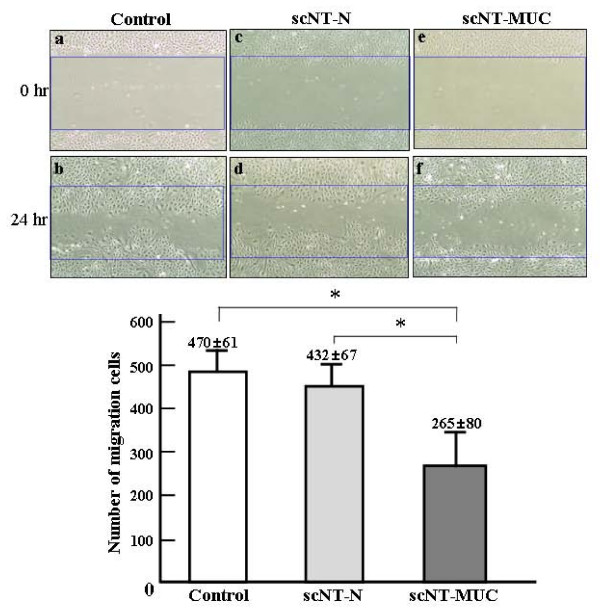
**Wound healing assay**. scNT-MUC-derived PUVECs (e and f) had significantly lower levels of cell migration than control (a and b) and scNT-N (c and d) PUVECs. The graph shows the number of cells that migrated into the wound 24 hr after wounding. The data were expressed as the mean value of cell numbers measured in individual control, scNT-N and scN-MUC with at least 3 independent experiments. * *P *< 0.05.

To determine whether the changes in angiogenesis-related gene expression that we observed earlier in scNT-MUC affected EC motility, we performed a migration assay using PUVEC cell lines from control, scNT-N and scNT-MUC piglets. PUVECs were grown to confluence and an area of the dish (the so-called wound) was scraped clear of cells by applying suction with a narrow tip (Figure 5-a, -c, and --5e). Cells were allowed to incubate for 24 hr, and then examined by microscopy. The migration of scNT-MUC-derived PUVECs was significantly impaired as compared to control and scNT-N-derived PUVECs (Figure 5-b, -d, and 5-f), with statistically significant differences in the numbers of migrating and proliferating cells (Figure 5).

**Figure 6:**
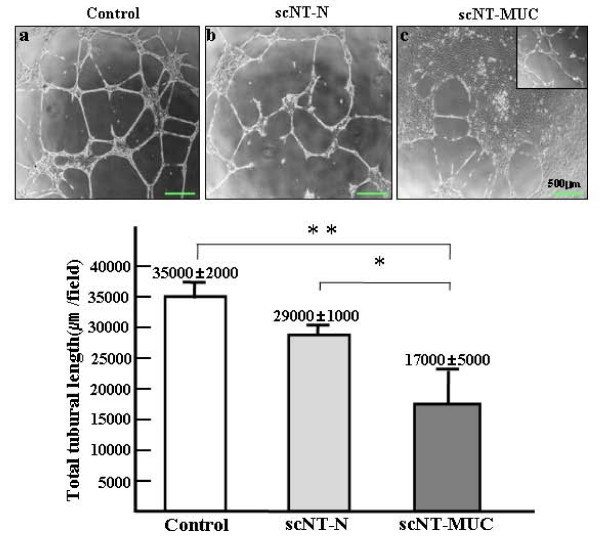
***In vitro *junction and tubule formation assay**. Control and scNT-N-derived PUVECs formed an extensive capillary-like network 24 hr after plating onto Matrigel (a and b), while scNT-MUC PUVECs formed a truncated and abnormal network (c). Cells were viewed by phase-contrast microscopy. Magnification, 40×. Tubule length was measured under a microscope and then average tubule length was plotted. The data were expressed as the mean value of tubule length measured in individual control, scNT-N and scN-MUC with at least 3 independent experiments. * *P *< 0.05, ** *P *< 0.01.

In addition to the migration and proliferation of ECs, proper vascularization requires vascular maturation, during which the rate of vascular sprouting is attenuated to prevent vascular collapse. Vascular maturation involves the recruitment of both perivascular and smooth muscle cells [28]. We next examined the ability of scNT-MUC-derived PUVECs to form EC junctions and tubules. Cells were plated on Matrigel, and allowed to incubate for 6 hr. Control and scNT-N PUVECs formed networks consisting of cellular aggregates (nodes), with branches of elongated strands of cells (Figure 6-a and 6-b). scNT-MUC-derived PUVECs also formed EC junctions, but the networks consisted of a small population of nodal cells, and tubular branches with an abnormal morphology (Figure 6-c). scNT-MUC-derived PUVECs also formed significantly shorter tubules than control and scNT-N PUVECs (Figure 6). These results suggested that decreased expression of angiogenesis-related genes in scNT-MUC results in defective PUVEC migration and tubule formation.

### scNT umbilical cords express low levels of glycolytic- and cell motility-associated proteins

**Figure 7:**
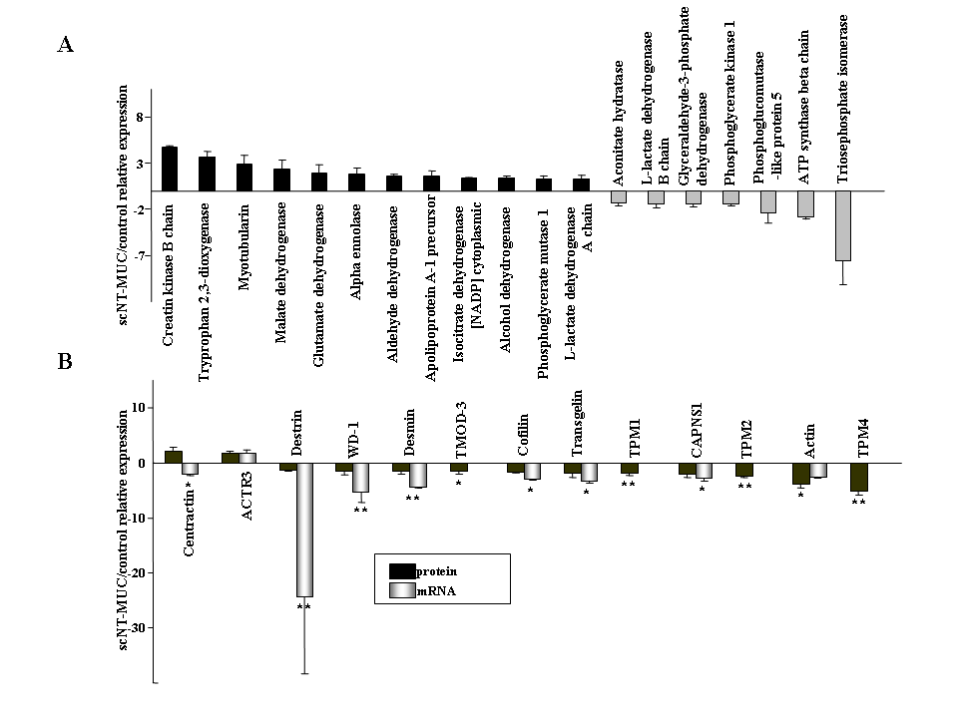
**Proteomic analysis of control and scNT-MUC**. A) 2-DE analysis of differentially expressed proteins in control and scNT-MUC. Several glycolytic enzymes and TCA-related enzymes were down- and up-regulated, respectively, in scNT-MUC relative to control. B) Analysis of the mRNA and protein expression levels of cell motility-related proteins in scNT-MUC relative to control umbilical cords. Real-time RT-PCR and 2-DE analyses were independently performed in triplicate for different samples and the data were expressed as the mean value of gene and protein expression measured in individual control and scNT-MUC. *p < 0.01; **p < 0.001.

The umbilical cords of rodents and large mammals, including humans, are glucose-dependent tissues that undergo limited mitochondrial respiration, and rely predominantly on the anaerobic conversion of glucose to lactate [29]. We compared the proteomes of scNT-MUC and control umbilical cords by 2-dimensional gel electrophoresis (2-DE) (see Additional file 3 and 4), and found that the expression levels of several glycolytic enzymes were lower in scNT-MUC (Figure 7A). For example, phosphoglucomutase-like protein 5, triosephosphate isomerase, phosphoglycerate kinase 1 and L-lactate dehydrogenase B chain were down-regulated by 2.4-, 7.5-, 1.3-, and 1.4-fold, respectively. By comparison, a number of enzymes involved in aerobic metabolism (TCA cycle) were up-regulated in scNT-MUC (Figure 7A and see Additional file 4). These results suggested that, unlike normal umbilical cords, scNT-MUC lack sufficient protection from oxidative damage due to the decreased expression of key glycolytic enzymes and antioxidant proteins. Several other classes of protein whose expression differed between control umbilical cords and scNT-MUC were identified, and included proteins involved in detoxification, chaperone activity, signal transduction and cytokine activity (see Additional file 4). Of note, several proteins involved in the regulation of the cytoskeleton and motility were differentially expressed in control umbilical cords and scNT-MUC.

We performed real-time RT-PCR to validate some of the data derived from 2-DE analysis, and to investigate the relationship between the mRNA and protein expression levels of cytoskeletal- and motility-related proteins. As shown in Figure 7B, the mRNA levels of destrin, WD-repeat protein 1 (WD-1), desmin, ubiquitous tropomodulin 3 (TMOD3), cofilin, transgelin, tropomyosin 1 alpha (TPM-1), calpain small subunit (CAPNS1), tropomyosin 1 beta (TPM-2), actin and tropomyosin alpha 4 chain (TPM-4) were significantly lower in scNT-MUC (*p *< 0.05) than in control. In particular, there was a pronounced difference in the mRNA expression level of destrin (Figure 7B, *p *< 0.01). When we directly compared protein and mRNA expression patterns, with the exception of alpha-centractin, all cytoskeletal and motility-related proteins exhibited similar patterns of down-regulation at both the protein and mRNA levels in scNT-MUC (Figure 7B). Thus, mRNA expression levels in the umbilical cord closely mirrored protein expression levels. The exception was alpha-centractin, which was up-regulated at the protein level, and down-regulated at the mRNA level in scNT-MUC. These results suggested that alterations in the expression of proteins involved in the cytoskeleton and motility lead to defects in placental EC migration and tubule formation. Defects in EC signal transduction and function could lead to the characteristic disorganization of myofibers in scNT-MUC-derived placentas.

### Up-regulation of apoptotic proteins and down-regulation of oxidative repair proteins in scNT-MUC

A number of proteins involved in apoptosis and cell cycle signaling were consistently altered in scNT-MUC relative to control umbilical cords (see Additional file 4). In particular, proteins in two major categories of apoptosis-related proteins, lipid-binding and oxireductase activity, were frequently altered in scNT-MUC. The lipid-binding apoptosis-related proteins Annexin A1, A2, and A5 were up-regulated in scNT-MUC. Annexins are structural proteins that bind to phospholipids in a Ca^+2^-dependent manner, and are well-characterized apoptosis biomarkers [30]. The expression levels of lamin A and HSP27 were also up-regulated in scNT-MUC as compared to controls. Lamin is cleaved by members of the interleukin-converting enzyme family during apoptosis [31], and HSP27 (and HSP71) induces apoptosis through the activation of the caspase cascade [32]. These results were indicative of increased levels of apoptosis in scNT-MUC. The oxireductase activity-related proteins peroxiredoxin (Prx)-2 and -4 and Cu/Zn superoxide dismutase (SOD) were significantly down-regulated in scNT-MUC, which suggested that damaging reactive oxygen species (ROS) accumulate in scNT-MUC and contribute to apoptosis [33].

**Figure 8:**
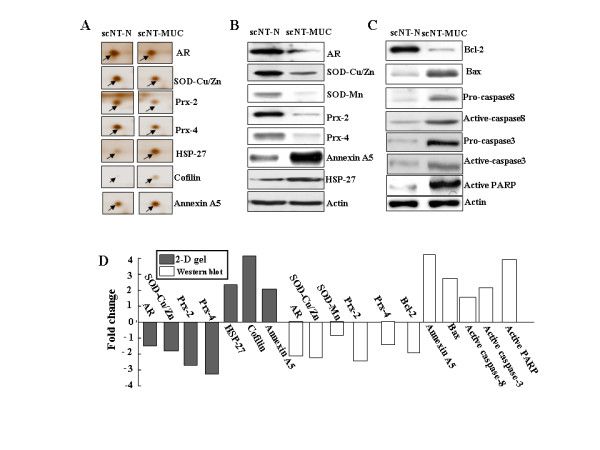
**Proteomic analysis of scNT-N and scNT-MUC by 2-DE and Western blot**. A) Representative up- and down-regulated proteins from scNT-N and scNT-MUC are shown. B and C) Western blot analysis of ROS- and apoptosis-related proteins in scNT-N and scNT-MUC. The amount of total protein loaded per lane was 15-30 μg; β-actin was analyzed as a loading control. D) Fold changes of differentially expressed proteins in scNT-N and scNT-MUC by 2-DE gel or Western blotting.

To identify the minimal repertoire of molecules involved in the development of MUC while also controlling for variation due to scNT, we carried out a comparative proteomic analysis of scNT-N and scNT-MUC using 2-DE followed by MALDI-TOF/TOF mass spectrometry. We identified 70 protein species that appeared to be differentially expressed in scNT and scNT-MUC (see Additional file 5 and 6). Among them, 35 were present at higher levels in scNT-MUC, while 25 were present at lower levels as compared to scNT-N. Additional file 6 summarizes the main properties of 40 of the proteins identified that were differentially regulated in scNT-MUC and scNT-N. The expression of apoptosis-related proteins (annexin A2, A5, and HSP 27) was up-regulated, whereas the expression of cell motility and structural proteins (heat-shock 20 kDa like-protein p20, transgelin, tubulin-specific chaperone A, LIM and SH3 protein 1, destrin), and detoxification proteins [Prx-2, -4, SOD-Cu/Zn, aldose reductase (AR)] was down-regulated (Figure 8A and 8D). Western blot analysis confirmed the up-regulation of annexin A5 and HSP27, and the down-regulation of AR, SOD-Cu/Zn, SOD-Mn, Prx-2 and Prx-4 in scNT-MUC (Figure 8B and 8D).

Given the apparent increase in the expression of apoptosis-related proteins in scNT-MUC, we next examined the expression of several proteins that are key factors in programmed cell death. The level of expression of the anti-apoptotic protein Bcl-2 was lower in scNT-MUC than scNT-N, whereas Bax, active poly (ADP-ribose) polymerase (PARP) and pro/active caspases 3 and 8 were significantly up-regulated (Figure 8C and 8D). PARP is an important substrate of caspase-3 that is cleaved from a 112-kDa fragment into an 85-kDa fragment upon caspase-3 activation [31,32,34]. These results provided additional evidence of active apoptosis in scNT-MUC. While the elimination of unwanted cells during development is essential, inappropriate or elevated apoptosis in the umbilical cord can result in destruction or weakening of the tissue. The umbilical cord is essential during development, as it provides nutritional, endocrine and immune support to the fetus. When these functions are compromised due to elevated apoptosis, low placental weights and low birth weights can ensue.

### TUNEL analysis of organs from scNT-MUC piglets

**Figure 9:**
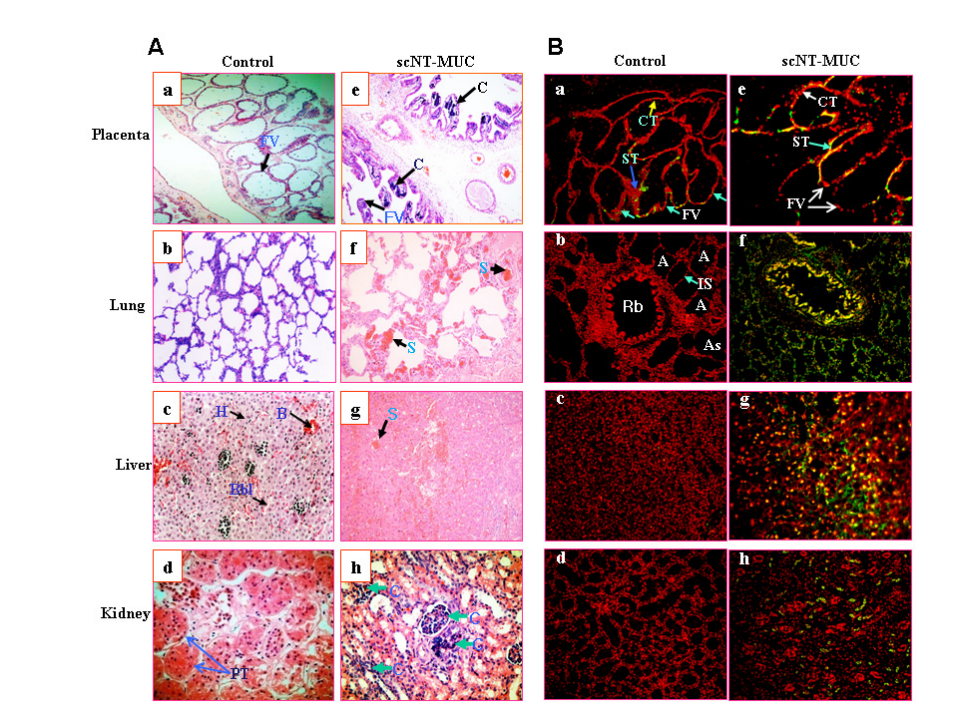
**Analysis of organs derived from control and scNT-MUC piglets**. A) Microscopic analysis revealed evidence of severe calcification in scNT-MUC-derived placenta (e) and kidney (h). Lung (f) and liver (g) sections derived from scNT clones with MUC showed abnormal stromal cell proliferation, peliosis in the sinusoidal space and tubular degeneration. B) De-waxed paraffin sections of placenta, lung, liver and kidney from control (n = 3) and scNT-MUC (n = 6) piglets were subjected to a TUNEL assay. Increased apoptosis was evident in several organs (e-h) derived from scNT clones with MUC, while few TUNEL-positive cells were observed in control organs (a-d). Apoptosis is indicated by brown or green coloration. CT, cytotrophoblast; ST, syncytotrophoblast; A, alveoli; As, alveolar sac; B, blood vessels; C, calcification; Fv, floating villi; H, hepatocytes; IS, interalveolar septum; Bbl, border between lobules; Rb, respiratory bronchiole; PT, proximal tubules, S, stromal cell. Magnification, 200×.

To investigate the relationship between MUC and fetal malformation, we examined several postnatal fetal organs by microscopy (Figure 9A). We observed severe congestion in the lung and liver of scNT-MUC piglets (Figure 9A-f and 9-g). The affected tissues had a prominent red color due to engorgement with oxygenated blood and exhibited severe peliosis. There was also evidence of abnormal stromal cell proliferation and tubular degeneration in the lung and liver (Figure 9A-f and 9-g, arrows). Of note, distinct areas of calcification were detected in the placenta and kidney (Figure 9A-e and 9-h, arrowheads). We also measured the level of apoptosis in control and scNT-MUC-derived placenta, lung, liver and kidney using the TUNEL assay (Figure 9B). Whereas apoptotic cells were barely detectable in control placentas (Figure 9B, a-d), there was a marked elevation in apoptosis in organs derived from scNT-MUC clones (Figure 9B, e-h). Apoptosis in scNT-MUC placentas was restricted primarily to cytotrophoblast and syncytotrophoblast cells, with the exception of a small sub-population of cells attached to the basal membrane of the villi (Figure 9B-e and Figure 3, lower right panel).

Recent studies from our laboratory suggest that the low success rate of scNT cloning is due to placental abnormalities rather than to cumulative genomic damage [3,7,11,12]. The data from the current study are consistent with this hypothesis, and indicate that small placentas derived from scNT-MUC inflict chronic pressure on the developing fetus, resulting in compromised organ development. Thus, MUC contribute to placental insufficiency, and ultimately influence fetal development, malformation and birth rates.

## Conclusion

Our observations suggest that due to MUC, the blood flow may be reduced between placenta and fetus, resulting in an increase in apoptosis in the umbilical cord. Because the umbilical cord plays a fundamental role in transporting metabolites between mother and fetus, MUC also contribute to placental insufficiency by preventing the removal of harmful materials from fetal circulation. MUC might also promote hypoxia and the accumulation of CO_2_, while diminishing O_2 _levels in fetal organs, stimulating apoptosis in the developing fetus. Thus, the functional consequences of MUC in scNT-derived animals are potentially severe, and include not only placental insufficiency, fetal abnormalities and mortality, but also fetal malformations, preterm birth, and low birth weight. Our data suggest that these effects are due to specific molecular defects that lead to the development of HUA and/or placental insufficiency. In summary, the results of the current study provide several clues to a better understanding of the molecular mechanisms of angiogenesis during umbilical cord development, as well as a robust model for the study of HUA syndrome in humans.

## List of abbreviations

AR: aldose reductase; Ang: Angiopoietin; CAPNS1: calpain small subunit; 2-DE: 2-dimensional gel electrophoresis; EC: Endothelial cell; HUAs: hypoplastic umbilical arteries; ILSM: inner longitudinal smooth muscle; MUC: malformed umbilical cords; OCSM: outer circular smooth muscle; PARP: poly (ADP-ribose) polymerase; PECAM-1: platelet endothelial cell adhesion molecule-1; Prx: peroxiredoxin; ROS: reactive oxygen species; PUVEC: porcine umbilical vein-derived endothelial cells; scNT: somatic cell nuclear transfer; SOD: superoxide dismutase; SUA: single umbilical artery; TMOD3: tropomodulin 3; TPM-1: tropomyosin 1 alpha; TPM-2: tropomyosin 1 beta; TPM-4: tropomyosin alpha 4 chain; TUNEL: TdT-mediated dUTP-X Nicked End labeling; VEGF: vascular endothelial growth factor; WD-1: WD-repeat protein 1

## Authors' contributions

HHP, SSP performed the experiments in Figure 1 and 3. YJC, KCH, SKC carried out the scNT, embryo transfer and molecular biology. TK, CP, HTL and HGS performed protein studies. SBP and SH performed the experiments in Figure 4, 5, 6, and 9. JYP and both JHKs carried out molecular biology, protein studies and conceived, designed and wrote the manuscript. All authors read and approved the final manuscript.

## Supplementary Material

Additional file 1 **Table s1**. Click here for file

Additional file 2 **Materials and Methods for Supplementary Figures S1 and S2**. Click here for file

Additional file 3 **Figure s1**. Click here for file

Additional file 4 Table s2. Click here for file

Additional file 5 **Figure s2**. Click here for file

Additional file 6 Table s3. Click here for file
