# Supplementary material for: Comparative proteomic analysis of malformed umbilical cords from somatic cell nuclear transfer-derived piglets: implications for early postnatal death
Source: BMC Genomics. 2009 Nov 5;10:511. doi: 10.1186/1471-2164-10-511 (PMC2783166; doi:10.1186/1471-2164-10-511)
Supplement: Additional file 1 — Table s1. [file 1471-2164-10-511-S1.doc]

**Supplementary Table 1.** RT-PCR primer sequences.

| Gene | Description | Primer (5’🡪3’) | Length(bp) | Acc. No. |
| --- | --- | --- | --- | --- |
| VEGF | Vascular endothelial growth factor | F: ccttgctgctctacctccac  R: atggcgatgttgaactcctc | 271 | NM_214084 |
| VEGFR1 | VEGF receptor 1 | F: agagcgacgtgtggtcctac  R: tccacaaatcttggcctttc | 211 | AJ245445 |
| VEGFR2 | VEGF receptor 2 | F: gagtggctctgaggaacgag  R: acacaactccatgctggtca | 209 | BQ603967 |
| Ang-1 | Angiopoietin 1 | F: atcttaatgggggaggttgg  R: gctctgttttcctgctgtcc | 296 | NM_213959 |
| Ang-2 | Angiopoietin 2 | F: cactggctgggaaatgagtt  R: agcctcctgtgagcatctgt | 277 | NM_213808 |
| Tie-2 | Tyrosine kinase Tie2 | F: gatggtggagaagcctttca  R : tgcacgcagagctcatattc | 261 | AF251494 |
| Desmin | Desmin | F: taaagccagagacccttctg R: ctgaagactctgccctttct | 450 | NM_001001535 |
| Transgelin | Smooth muscle protein 22-alpha | F: aagggtccttcctatggcat  R: ccatcattcttggtcactgc | 399 | NM_001046149  BC093050 |
| Centractin | Centrosome-associated actin homolog | F: catggagtcctacgatgtga  R: gcacattgaaggtctcgaag | 404 | NM_016860  NM_005736 |
| Actin | Alpha-actin, smooth muscle, aorta | F: atcatcaccactgggacga  R: cagtcaggatcttcatgagg | 339 | NM_001613 |
| GAPDH | Glyceraldehyde-3-phosphate dehydrogenase | F: aagtggacattgtcgccatc  R: tcacaaacatgggggcatc | 318 | X94251 |
| Cofilin | Cofilin | F: gcaatcccttcaccccactt  R: cggttagaagttggcagcat | 239 | M20866 |
| ACTR3 | ARP3 actin-related protein 3 homolog | F: ttcaaccatgttcagggact  R: aacactggattgtgacgaca | 270 | NM_023735  NM_005721 |
| WD-1 | WD repeat protein 1,  Actin interacting protein | F: gaggaagtgcttcagtgtcg  R: cgtacaccatcatgtccatg | 360 | NM_011715 |
| CAPNS1 | Calpain samall subunit  Calpain I light subunit | F: ctgtactgctgtgcttgtga  R: tccaggttagagcagaccaa | 259 | NM_214318 |
| Destrin | Actin-depolymerizing factor | F: ttggcccaaattcttgagtc  R: agctcagcgctggttctaag | 173 | D90053 |
| TPM-1 | Tropomyosin-1 | F: ctctgaacagacgcatccaa  R: cctgagcctccagtgacttc | 372 | NM_001097483 |
| TPM-2 | Tropomyosin-2 | F: aaactggagcaggctgaaaa  R: gttcgcagttcctcttccag | 398 | DQ306707 |
| TPM-4 | Tropomyosin-4 | F: gcgagagaggaatgaaggtg  R: agcgacttcaggttgttggt | 253 | AF087679 |
| TMOD3 | Ubiquitous tropomodulin 3 | F: gcctcctgttgtgatgtca  R: aggtgcaacagcaattctg | 347 | NM_214294 |
| Tubulin | Tubulin alpha | F: gaccacaagtttgacctgat  R: gggcttaaaggaatggtaat | 220 | DQ225365 |
