# Supplementary material for: Comparative proteomic analysis of malformed umbilical cords from somatic cell nuclear transfer-derived piglets: implications for early postnatal death
Source: BMC Genomics. 2009 Nov 5;10:511. doi: 10.1186/1471-2164-10-511 (PMC2783166; doi:10.1186/1471-2164-10-511)
Supplement: Additional file 2 — Materials and Methods for Supplementary Figures S1 and S2. [file 1471-2164-10-511-S2.doc]

**Materials and Methods for Supplementary Figures 1 and 2.**

***2-DE and protein identification***

2-DE and spot analysis were performed as previously described [1,2], with slight modifications. Umbilical cords were solubilized in lysis buffer containing 7 M urea, 2 M thiourea, 4% w/v CHAPS, 40 mM DTT and 0.5% Pharmalyte pH 4-7. Insoluble material was removed by centrifugation. IPG strips (17 cm, pH 4-7, Bio-Rad, Hercules, CA, USA) were rehydrated overnight in 300 μL of lysate containing 500 μg of protein. Isoelectric focusing was performed using a Protein IEF Cell (Bio-Rad). The focused strips were then equilibrated by incubating them first in equilibration solution (6 M urea, 30% v/v glycerol, 2% w/v SDS, 50 mM Tris-Hcl, pH 8.8) containing 1% w/v DTT for 15 min, followed by a second incubation in 2.5% w/v iodoacetamide in the same equilibration solution for 15 min. 2-DE was performed using 0.7 cm thick, 18Ⅹ18 cm linear gradient gels (7.5-17.5%) in a Protein ∏ xi 2-D Cell apparatus (Bio-Rad). The gels were stained with silver or Coomassie Brilliant Blue G250 to visualize proteins. Images of stained gels were digitized with a densitometer (Versa Doc Imagin System 1000^TM^, Bio-rad). The density of spots was detected and counted by both automation and manual spot-detection, and statistically analyzed with PDQuest software (Version 7.1.1, Bio-Rad). Protein expression data from gels were normalized for the total density presented in gel images.

Target spots that were identified by PDQuest software (Bio-Rad) were excised from the gel, destained, and then subjected to in-gel digestion with bovine trypsin (Promega, Madison, WI, USA). Peptides were analyzed by MALDI-TOF mass spectrometry using a Voyager DE-STR MALDI-TOF mass spectrometer (Applied Biosystems, Framingham, MA, USA) in reflection mode, at a 20 kV accelerating voltage [1,2]. Database searches were performed using ProteinProspector ([http://propector.UCSF.edu](http://propector.UCSF.edu/)) and PROWL ([http://www.proteometrics.com](http://www.proteometrics.com/)).

***MALDI TOF/TOF analysis***

MALDI TOF/TOF analysis was performed in the Proteomics Facility Core of the Bio-Food and Drug Research Center at Konkuk University. 2-DE and spot analysis were performed as previously described [3,4].

**References**

1. [Park MR, Cho SK, Lee SY, Choi YJ, Park JY, Kwon DN, Son WJ, Paik SS, Kim T, Han YM, Kim JH](http://www.ncbi.nlm.nih.gov/entrez/query.fcgi?cmd=Retrieve&db=pubmed&dopt=Abstract&list_uids=15832370&query_hl=1&itool=pubmed_docsum): **A rare and often unrecognized cerebromeningitis and hemodynamic disorder: a major cause of sudden death in somatic cell cloned piglets**. *Proteomics* 2005, **5:**1928-1939.

2. Lee SY, Park JY, Choi YJ, Cho SK, Ahn JD, Kwon DN, Hwang KC, Kang SJ, Paik SS, Seo HG, Lee HT, Kim JH: **Comparative proteomic analysis associated with term placental insufficiency in cloned pig**. *Proteomics* 2007, **7:**1303-1315.

3. [Lee CK, Park HJ, So HH, Kim HJ, Lee KS, Choi WS, Lee HM, Won KJ, Yoon TJ, Park TK, Kim B](http://www.ncbi.nlm.nih.gov/pubmed/17099934?ordinalpos=12&itool=EntrezSystem2.PEntrez.Pubmed.Pubmed_ResultsPanel.Pubmed_RVDocSum): **Proteomic profiling and identification of cofilin responding to oxidative stress in vascular smooth muscle**. *Proteomics* 2006, **6:**6455-6475.

4. [Lee CK, Kim HJ, Lee YR, So HH, Park HJ, Won KJ, Park T, Lee KY, Lee HM, Kim B](http://www.ncbi.nlm.nih.gov/pubmed/17556052?ordinalpos=7&itool=EntrezSystem2.PEntrez.Pubmed.Pubmed_ResultsPanel.Pubmed_RVDocSum): **Analysis of peroxiredoxin decreasing oxidative stress in hypertensive aortic smooth muscle**. *Biochem Biophys Acta* 2007, **1774:**848-855.
