# Supplementary material for: Comparative proteomic analysis of malformed umbilical cords from somatic cell nuclear transfer-derived piglets: implications for early postnatal death
Source: BMC Genomics. 2009 Nov 5;10:511. doi: 10.1186/1471-2164-10-511 (PMC2783166; doi:10.1186/1471-2164-10-511)
Supplement: Additional file 3 — Figure s1. [file 1471-2164-10-511-S3.doc]

**Supplementary Figure 1.** Proteomic analysis of control and scNT-MUC. A) 1-dimensional gel electrophoresis of proteins from control and scNT-MUC. Arrows indicate candidate proteins **that appear to be** differentially expressed in control and scNT-MUC. B) 2-DE analysis. C) Relative intensity of differentially expressed genes. For details, see Supplementary Table 2.
