# Supplementary material for: Comparative proteomic analysis of malformed umbilical cords from somatic cell nuclear transfer-derived piglets: implications for early postnatal death
Source: BMC Genomics. 2009 Nov 5;10:511. doi: 10.1186/1471-2164-10-511 (PMC2783166; doi:10.1186/1471-2164-10-511)
Supplement: Additional file 4 — Table s2. [file 1471-2164-10-511-S4.doc]

**Supplementary Table 2. Differentially expressed proteins in control and scNT-MUC by MALDI-TOF MS.**

| Spot No. | ^1^Up/  Down | | | | | | Identified protein | Accession  No. | ^2^Mr  (Da)  Theor. | PI  Theor. | ^3^Mr  Exp. | PI  Exp. | ^4^Cov  (%) |
| --- | --- | --- | --- | --- | --- | --- | --- | --- | --- | --- | --- | --- | --- |
| Cell mobility or structure related protein | | | | | | | | | | | | | |
| 10 | -1.6 | | | | | | WD-repeat protein 1 | O88342 | 66407 | 6.11 | 66407 | 6.65 | 42 |
| 12 | -1.6 | | | | | | Desmin | P02540 | 53629 | 5.21 | 54.6 | 5.21 | 40 |
| 23 | -1.6 | | | | | | Tropomodulin 3 | Q9NYL9 | 39595 | 2.08 | 39595 | 2.08 | 30 |
| 24 | -2 | | | | | | Tropomyosin-1 | P58771 | 32681 | 4.69 | 32681 | 4.69 | 28 |
| 25 | -2.4 | | | | | | Tropomyosin-2 | P58776 | 32837 | 4.66 | 32837 | 4.66 | 32 |
| 30 | 1.7 | | | | | | ARP3 actin-related protein 3 homolog | Q99JY9 | 47357 | 5.61 | 47357 | 5.61 | 33 |
| 40 | -3.8 | | | | | | Actin | P62740 | 42009 | 5.2 | 42009 | 5.2 | 12 |
| 47 | -5.2 | | | | | | Tropomyosin-4 | P67936 | 28522 | 4.6 | 28522 | 4.6 | 27 |
| 56 | -2.1 | | | | | | Calpain small subunit | P04574 | 28069 | 5.0 | 28069 | 5.0 | 24 |
| 60 | 1.6 | | | | | | Myosin regulatory light chain 2 | P29269 | 30326 | 5.48 | 25.3 | 5.0 | 38 |
| 89 | -1.9 | | | | | | Transgelin | Q9TS87 | 20363 | 6.96 | 17.3 | 5.9 | 23 |
| 112 | -1.4 | | | | | | Destrin(actin-depolymerizing factor) | P60981 | 18506 | 8.1 | 18506 | 8.1 | 39 |
| 113 | -1.7 | | | | | | Cofilin, non-muscle isoform (cofilin-1) | P23528 | 18503 | 8.2 | 18503 | 8.2 | 39 |
| 114 | 2.1 | | | | | | Alpha-centractin | P61164 | 84614 | 6.2 | 84614 | 6.2 | 15 |
| Chaperone related protein | | | | | | | | | | | | | |
| 1 | -1.4 | | | | | | Endoplasmin precursor | Q29092 | 92471 | 4.75 | 92471 | 4.75 | 20 |
| 2 | -1.8 | | | | | | 78kDa glucose-regulated protein precursor | P11021 | 72333 | 5.07 | 72333 | 5.07 | 29 |
| 5 | 1.6 | | | | | | Heat shock congnate 71kDa | P19120 | 71240 | 5.49 | 71240 | 5.49 | 47 |
| 27 | -2.9 | | | | | | Protein disulfide isomerase A3 precursor | P30101 | 56783 | 5.99 | 56783 | 5.99 | 46 |
| 85 | 3.1 | | | | | | Heat shock protein beta-1(Hsp 27) | P42930 | 22893 | 6.12 | 22893 | 6.12 | 16 |
| 97 | -1.3 | | | | | | T-complex protein 1. beta subunit | P80314 | 57474 | 6.0 | 57474 | 6.0 | 22 |
| Detoxification related protein | | | | | | | | | | | | | |
| 61 | | -2.8 | | | | | Peroxiredoxin 2 | P52552 | 14175 | 4.7 | 14175 | 4.7 | 29 |
| 79 | | 1.9 | | | | | NG,,NG-dinethylarginine dimethylaminohydrolase | O95865 | 29644 | 5.66 | 29644 | 5.66 | 25 |
| 83 | | -2.5 | | | | | Peroxiredoxin 4 | Q13162 | 30540 | 5.86 | 30540 | 5.86 | 20 |
| 90 | | -2 | | | | | Superoxide dismutase [Cu-Zn] | P04178 | 15761 | 6.05 | 15761 | 6.05 | 33 |
| Signal transduction related protein | | | | | | | | | | | | | |
| 31 | | | 1.3 | | Rab GDP-dissociation inhibitor beta-2 | | | Q61598 | 50537 | 5.93 | 50537 | 5.93 | 43 |
| 42 | | | 6.9 | | Maspin precursor | | | P70124 | 42112 | 5.55 | 42113 | 5.55 | 50 |
| 48 | | | -1.6 | | 14-3-3 protein tau | | | P27348 | 27764 | 4.7 | 27764 | 4.7 | 53 |
| 49 | | | -1.5 | | 14-3-3 protein zeta/delta | | | P63104 | 27745 | 4.7 | 27745 | 4.7 | 53 |
| 52 | | | 6.2 | | 14-3-3 protein sigma | | | O77642 | 27713 | 4.75 | 27713 | 4.75 | 31 |
| 63 | | | 1.3 | | Rho GDP-dissociation inhibitor 1 | | | P19803 | 23422 | 5.12 | 23433 | 5.12 | 38 |
| 75 | | | -1.4 | | Guanin nucleotide-binding protein beta | | | P54311 | 37377 | 5.6 | 37377 | 5.6 | 23 |
| 109 | | | 1.8 | | Guanin nucleotide-binding protein beta subunit 2 | | | P68040 | 35077 | 7.6 | 35077 | 7.6 | 39 |
| Calcium/phospholipid binding protein | | | | | | | | | | | | | |
| 44 | | | | 5.8 | | | Annexin A1 | P19619 | 38,769 | 6.4 | 38,769 | 6.4 | 27 |
| 46 | | | | 5 | | | Annexin A2 | P04272 | 38,612 | 6.9 | 38,612 | 6.9 | 38 |
| 53 | | | | 2 | | | Annexin A5 | P81287 | 36074 | 4.9 | 36074 | 4.7 | 40 |
| Metabolism related protein | | | | | | | | | | | | | |
| 14 | | | -2.8 | | ATP synthase beta chain | | | P00829 | 56284 | 5.15 | 56284 | 5.1 | 38 |
| 32 | | | 1.6 | | Aldehyde dehydrogenase | | | P49189 | 53533 | 6.0 | 53533 | 6.0 | 14 |
| 34 | | | 1.8 | | Alpha ennolase | | | P04764 | 47277 | 6.44 | 47277 | 6.44 | 26 |
| 39 | | | 4.7 | | Creatin kinase B chain | | | Q04447 | 42714 | 5.4 | 42714 | 5.4 | 23 |
| 41 | | | 2.3 | | Malate dehydrogenase | | | P11708 | 36454 | 6.16 | 36454 | 6.16 | 19 |
| 77 | | | -1.4 | | L-lactate dehydrogenase B chain | | | P00336 | 36613 | 5.6 | 36613 | 5.6 | 28 |
| 98 | | | -2.4 | | Phosphoglucomutase-like protein 5 | | | Q15124 | 55613 | 6.8 | 55613 | 6.8 | 27 |
| 100 | | | 1.9 | | Glutamate dehydrogenase | | | P00366 | 61512 | 7.2 | 61512 | 7.2 | 18 |
| 104 | | | 3.7 | | Tryprophan 2,3-dioxygenase | | | P48775 | 47872 | 6.5 | 47872 | 6.5 | 16 |
| 105 | | | 1.4 | | Isocitrate dehydrogenase [NADP] cytoplasmic | | | O75874 | 46660 | 6.5 | 46660 | 6.5 | 19 |
| 106 | | | -7.6 | | Triosephosphate isomerase | | | P17751 | 16713 | 6.9 | 16713 | 6.9 | 34 |
| 107 | | | 1.3 | | Phosphoglycerate mutase 1 | | | P18669 | 28804 | 6.7 | 28804 | 6.7 | 37 |
| 110 | | | 1.3 | | L-lactate dehydrogenase A chain | | | P00339 | 36619 | 8.2 | 36619 | 8.2 | 39 |
| 111 | | | -1.4 | | Glyceraldehyde-3-phosphate dehydrogenase | | | P00355 | 35836 | 8.5 | 35836 | 8.5 | 18 |
| 115 | | | 2.9 | | Myotubularin | | | Q9Y216 | 45437 | 7 | 45437 | 7 | 14 |
| 117 | | | 1.4 | | Alcohol dehydrogenase | | | P50578 | 36539 | 6.5 | 36539 | 6.5 | 27 |
| 118 | | | -1.4 | | Phosphoglycerate kinase 1 | | | Q7SIB7 | 44559 | 8 | 44559 | 8 | 32 |
| **Cytokine** | | | | | | | | | | | | | |
| 45 | | | -2.7 | | Septin2 | | | P42208 | 41526 | 6.1 | 41526 | 6.1 | 24 |
| 116 | | | -1.5 | | Septin 11 | | | Q9NVA2 | 49399 | 6.4 | 49399 | 6.4 | 21 |
| **Others** | | | | | | | | | | | | | |
| 7 | | | 2.2 | | Serum albumin precursor | | | P08835 | 69411 | 5.92 | 69411 | 5.92 | 54 |
| 43 | | | -3.8 | | Leukocyte elastase inhibitor | | | P80229 | 42516 | 6.0 | 42516 | 6.0 | 19 |
| 71 | | | -5 | | | NEFA-interactin nuclear protein NIP 30 | | Q9GZU8 | 28912 | 5.4 | 28912 | 5.4 | 22 |
| 73 | | | -1.9 | | | Eukaryotic translation initiation factor 3 subunit 2 | | Q9QZD9 | 36461 | 5.4 | 36461 | 5.4 | 28 |
| 76 | | | -1.4 | | | 60s acidic ribosomal protein PO | | Q95140 | 33058 | 5.54 | 33058 | 5.54 | 14 |
| 91 | | | -3.4 | | | Elongation factor 2 | | P58252 | 33058 | 6.4 | 33058 | 6.4 | 13 |
| 92 | | | | 1.7 | | | Lamin A | P49189 | 53533 | 6.0 | 53533 | 6.0 | 15 |
| 93 | | | | 1.4 | | | Serotransferrin | P09571 | 76969 | 6.9 | 76969 | 6.9 | 10 |
| 102 | | | | 1.8 | | | Elogation factor 1-gamma | Q29387 | 49625 | 6.2 | 49625 | 6.2 | 17 |
| 119 | | | | 1.7 | | | GTP-binding nuclear protein Ran | P49189 | 53533 | 6.0 | 53533 | 6.0 | 37 |

^1^The indication of “up/down” is calculated by comparing differentially expressed proteins of scNT-MUC with those in control.

^2^Theoretical molecular weight

^3^Experimental molecular weight

^4^Sequence coverage
