# Supplementary material for: Comparative proteomic analysis of malformed umbilical cords from somatic cell nuclear transfer-derived piglets: implications for early postnatal death
Source: BMC Genomics. 2009 Nov 5;10:511. doi: 10.1186/1471-2164-10-511 (PMC2783166; doi:10.1186/1471-2164-10-511)
Supplement: Additional file 5 — Figure s2. [file 1471-2164-10-511-S5.doc]

**Supplementary Figure 2.** Proteomic analysis of scNT-N and scNT-MUC. A) 1-dimensional gel electrophoresis of scNT-N and scNT-MUC. Arrows indicate proteins that appear to be differentially expressed in scNT-N and scNT-MUC. B) 2-DE analysis. C) Relative intensity of differentially expressed proteins. For details, see Supplementary Table 3.
