# Supplementary material for: Comparative proteomic analysis of malformed umbilical cords from somatic cell nuclear transfer-derived piglets: implications for early postnatal death
Source: BMC Genomics. 2009 Nov 5;10:511. doi: 10.1186/1471-2164-10-511 (PMC2783166; doi:10.1186/1471-2164-10-511)
Supplement: Additional file 6 — Table s3. [file 1471-2164-10-511-S6.doc]

**Supplementary Table 3.** Differentially expressed proteins in scNT-N and scNT-MUC as identified by MALDI-TOF/TOF MS.

| Spot No. | ^1^Up/  Down | Identified protein | ^2^Accession No./database | ^3^Score | ^4^SC(%) | MW(Da)/PI | Known Function. |
| --- | --- | --- | --- | --- | --- | --- | --- |
| 0104 | -1.66 | Myosin catalytic light chain LC17a | 89245/NC  P60662/SP | 186 | 22%/NC  22%/SP | 16789/  4.56 | Regulatory light chain of myosin |
| 1007 | +1.75 | Galectin-1 | 47716872  /NC | 48 | 8%/NC | 14590/  5.07 | Sugar binding |
| 1013 | -1.52 | Galectin-1 | 47716872  /NC  P48538/SP | 339 | 37%/NC  8%/SP | 14590/  5.07 |
| 1102 | +4.20 | Myosin regulatory light chain, smooth muscle | 284569/NC  P29269/SP | 298 | 24%/NC  24%/SP | 19683/  4.80 | Regulation of smooth muscle contraction |
| 1123 | -6.77 | Myosin regulatory light chain, smooth muscle | 284569/NC  P29269/SP | 227 | 24%/NC  24%/SP | 19683/  4.80 |
| 1127 | -3.38 | Myosin regulatory light chain, smooth muscle | 284569/NC  P29269/SP | 56 | 5%/NC  5%/SP | 19683/  4.80 |
| 1128 | -1.77 | Myosin | 228542/NC  P29269/SP | 51 | 12%/NC  5%/SP | 19683/  4.80 |
| 1306 | +1.59 | Collagen alpha 1(I) chain precursor | 115269/NC  Q9XSJ7/SP | 76 | 1%/NC  1%/SP | 138799/5.66 | Protein binding |
| 4213 | +2.75 | Heat shock protein 27kDa | 50916342  /NC  P42929/SP | 57 | 8%/NC  4%/SP | 14211/  5.94 |
| 5302 | +1.63 | Type I collagen | 30102/NC  Q28668/SP | 69 | 8%/NC  6%/SP | 41496/  7.79 |
| 4104 | -2.42 | Heat-shock 20 kDa like-protein p20 | O14558/SP | 68 | 15%/SP | 17125/  5.95 |
| 1307 | +5.76 | Annexin A5 | 4139953/NC P14668/SP | 24 | 7%/NC  8%/SP | 35.5/4.9 | Apoptosis |
| 1317 | -8.67 | Peroxiredoxin-4 | 37590233  /NC Q63716/SP | 24 | 88%/NC | 31.0/  6.18 | Antioxidant activity |
| 3110 | -14.09 | Peroxiredoxin -2 | 34849738/  NC  P35704/SP | 17 | 8%/NC  7%/SP | 21.7/5.3 |
| 5101 | -2.63 | Superoxide dismutase | 66364/NC  P04178/SP | 103 | 15%/NC  26%/SP | 15751/  6.04 |
| 5104 | -9.14 | Smooth muscle protein 22-alpha | 2984713/NC  Q01995/SP | 92 | 40%/NC  18%/SP | 10099/  4.93 | Cytoskeleton Organization |
| 5315 | -5.62 | Annexin A2 | 37590785  /NC Q07936 /SP | 23 | 17%/NC  23%/SP | 38.5/7.5 |
| 6103 | -3.09 | Smooth muscle protein SM22 homolog | 543113/NC  Q01995/SP | 166 | 36%/NC  31%/SP | 19326/  6.96 |
| 6216 | -2.86 | Smooth muscle protein | 177175/NC  Q01995/SP | 54 | 11%/NC  11%/SP | 22461/  8.56 |
| 6217 | -12.96 | Smooth muscle protein | 177175/NC  Q01995/SP | 67 | 15%/NC  24%/SP | 22461/  8.56 |
| 4106 | -29.38 | Transgelin | Q9TS87/SP | 59 | 19%/SP | 20325/  6.96 | Actin binding |
| 6402 | +2.18 | LIM and SH3 protein 1 | 5453710/NC  Q14847/SP | 99 | 13%/NC  17%/SP | 29698/  6.61 |
| 5103 | +4.47 | Similar to Cofilin,  non-muscle isoform | 55636489  /NC  P10668/SP | 33 | 6%/NC  15%/SP | 38992/  10.11 | Actin polymerization |
| 7106 | -3.33 | Similar to destrin | 55651119  /NC  P60982/SP | 50 | 15%/NC  18%/SP | 18493/  8.06 |
| 7202 | +3.19 | Hemoglobin beta chain | 5542425/NC  P02067/SP | 422 | 49%/NC  38%/SP | 16025/  6.76 | Oxygen transporter activity |
| 8203 | +305.28 | Hemoglobin alpha chain | 70237/NC  P01965/SP | 39 | 10%/NC  10%/SP | 15030/  8.76 |
| 8206 | +114.62 | Hemoglobin alpha chain | 70237/NC  P01965/SP | 190 | 28%/NC  34%/SP | 15030/  8.76 |
| 8209 | +6.05 | Hemoglobin alpha chain | 70237/NC  P01965/SP | 222 | 34%/SP | 15030/  8.76 |
| 9111 | +4.2 | Hemoglobin beta chain | 5542425/NC  P02067/SP | 395 | 42%/NC  31%/SP | 16025/  6.76 |
| 4103 | +2.59 | Hemoglobin beta chain | P02067/SP | 50 | 20%/SP | 16024/  7.25 |
| 2205 | +1.56 | Heme-binding protein | 51847749  /NC | 168 | 28%/NC | 21056/  5.67 |
| 4409 | -1.65 | Aldose reductase | 48374071  /NC  P80276/SP | 46 | 5%/NC  5%/SP | 35844/  5.89 | Carbohydrate metabolism  Or  Antioxidant activity |
| 6206 | +2.61 | Carbonate dehydratase (EC 4.2.1.1) II | 68286/NC  P00921/SP | 64 | 5%/NC  5%/SP | 29322/  6.73 | Carbonate dehydratase activity |
| 4108 | -4.21 | Tubulin-specific chaperone A | 1711661/NC | 22 | 9%/NC | 12545/  5.25 | Chaperone binding  unfolded protein binding |
| 4204 | -1.70 | Cytidylate kinase | 1085437/NC  Q29561/SP | 28 | 4%/NC  4%/SP | 22265 | Cytidylate kinase activity |
| 4406 | +2.06 | Serine proteinase inhibitor member 5 | 16923954  /NC | 85 | 5%/NC | 42037/  5.78 | Serine-type endopeptidase inhibitor activity |
| 5004 | +1.58 | Putative transthyretin | 975233/NC  P50390/SP | 42 | 8%/NC  8%/SP | 15782/  6.34 | Central nervous system development |
| 5102 | +1.52 | LP2/NC  fatty acid-binding protein, epidermal | 1293786/NC  P55052/SP | 43 | 10%/NC  17%/SP | 15049/  7.57 | Transporter activity |
| 6203 | +1.80 | Complement factor D precursor | P51779/SP | 16 | 4%/NC | 27746/  6.59 | Complement factor D activity |
| 6205 | +2.89 | Blvrb protein | 13879286  /NC  P30043/SP | 277 | 31%/NC  12%/SP | 22183/  6.49 | Biliverdin reductase activity |

^1^The indication of “up/down” is calculated by comparing differentially expressed proteins of scNT-MUC with those in scNT-N.

^2^NC, NCBI; SP, SWISS-PROT

^3^Significant difference in MASCOT

^4^Sequence coverage
